# Maternal and Perinatal Outcome in Pregnancy Complicated by Intrahepatic Cholestasis

**DOI:** 10.7759/cureus.28512

**Published:** 2022-08-28

**Authors:** Manisha Jhirwal, Charu Sharma, Shashank Shekhar, Pratibha Singh, Satya Prakash Meena, Priyanka Kathuria, Apoorva Tak

**Affiliations:** 1 Obstetrics and Gynaecology, All India Institute of Medical Sciences Jodhpur, Jodhpur, IND; 2 General Surgery, All India Institute of Medical Sciences Jodhpur, Jodhpur, IND

**Keywords:** pruritus, pregnancy, lft, ihcp, fetomaternal outcome, bile acids

## Abstract

Introduction

Intrahepatic cholestasis of pregnancy (IHCP) is characterized by pruritus of the hand and sole with abnormal liver function test and bile acid metabolism. IHCP occurs in the second and third trimesters of pregnancy and usually resolves after delivery. The overall prevalence is about 1.2 to 1.5%. This study was conducted to assess the fetomaternal outcome according to maternal serum bile acids levels and its correlation with liver function tests in patients with IHCP.

Material and methods

This ambispective observational study was conducted in the department of Obstetrics and Gynecology (OBG) for two years at AIIMS Jodhpur, Rajasthan. It included all the pregnant women attending the outpatient department of OBG with the complaint of pruritis in the palm and sole after 28 weeks of pregnancy and diagnosed with intrahepatic cholestasis of pregnancy after investigations.

Results

Only 152 patients were diagnosed with IHCP out of 4,148 deliveries, with a prevalence of 3.6%. Among these, 140 (92.11%) had mild IHCP, 10 (6.58%) had moderate IHCP and two (1.32%) had severe IHCP. There was a significant difference between the birth weight in mild, moderate and severe IHCP (P-value 0.004). About 12.5% (n=19) of patients had meconium-stained liquor during delivery. Two patients (1.32%) with moderate IHCP had intrauterine fetal death in the third trimester, and 6.58% (n=10) neonates were kept on continuous positive airway pressure.

Conclusions

IHCP is associated with adverse fetal outcomes like spontaneous or iatrogenic preterm delivery, low birth weight, increase in the rate of lower section cesarean section (LSCS) and intrauterine death of a fetus. A significant correlation found between raised bile acid levels and variables of liver function test, hence cost-effectiveness and feasibility of liver function test (LFT) should be considered for the management of IHCP.

## Introduction

Intrahepatic cholestasis of pregnancy (IHCP) is a pregnancy-specific liver disease characterized by pruritus without any skin rash, abnormal liver function, and altered bile acid metabolism. The incidence is about 1.2 to 1.5% of cases of pregnancy [1]. IHCP cases are almost double in Asian women compared to European women. The highest incidence of IHCP (4%) was found in indigenous women from Chile and Bolivia [2]. Increased frequency of pruritus at night, involving the palms and soles without any skin lesion is particularly suggestive of IHCP. The serum bile acid level is sensitive and specific markers for IHCP. Serum aminotransferases more than two times of normal level and elevated alkaline phosphatase (ALP) levels are seen in IHCP but it is not specific for cholestasis. Total bilirubin concentrations rarely exceed 5 mg/dl [3].

Maternal bile acids get accumulated in the fetus and amniotic fluid by crossing the placental barrier, which carries significant risk for the fetus. The maternal complication associated with IHCP has increased risks of preterm prelabour rupture of membrane (PT PROM), severe pruritus with dyslipidemia and deranged coagulation profile, post-partum hemorrhage and operative delivery [4].

In the north Indian population, there is a lack of data indicating the association of various risk factors for IHCP with feto-maternal outcomes and the correlation of maternal serum bile acid with variables of liver function test. Hence, this study was done to assess feto-maternal outcomes in association with the severity of IHCP so that we can improve obstetrical outcomes without jeopardizing the perinatal outcomes, in addition, to find out the correlation of liver function test (LFT) with serum bile acids (BA) in patients with IHCP.

## Materials and methods

Study design and setting

This ambispective observational study was conducted in the department of Obstetrics and Gynecology (OBG) for two years (from 01 January 2019 to 01 January 2021) at All India Institute of Medical Sciences Jodhpur, Rajasthan, India.

Study population

This study included all the pregnant women with the complaint of pruritis in the palm and sole after 28 weeks of pregnancy and diagnosed with intrahepatic cholestasis of pregnancy based on abnormal liver function test and bile acid levels.

Study procedure

The diagnostic criteria used for IHCP were clinically evident unexplained pruritus especially in palms, soles without skin lesion with increased intensity at night and abnormal transaminase enzyme level of greater than twice the normal value and with serum bile acids more than 10 micromole/L. All singleton pregnant women beyond 28 weeks period of gestation (POG) who were diagnosed with IHCP and consented to participate were enrolled in the study. The exclusion criteria for the study were dermatological conditions leading to pruritus, viral hepatitis, HELLP (Haemolysis, elevated liver enzymes, low platelet count) syndrome, acute fatty liver of pregnancy and obstructive jaundice.

A detailed history was taken regarding age, parity, obstetric history, family history of diabetes, hypertension or IHCP and any recent change in drug intake. An obstetric examination was done. The dermatological cause of pruritis was ruled out by an expert opinion. Routine antenatal investigations with liver function tests and serum bile acid tests (fasting) were collected [5].

All patients were subsequently treated with tablet ursodeoxycholic acid (UDCA) 10-15 mg/kg/day in divided doses according to the level of serum bile acid. Liver enzymes were tested weekly/biweekly till delivery. All enrolled patients were clinically monitored and followed up in high-risk antenatal clinics weekly [6]. Fetal surveillance was done by non-stress test, modified biophysical profile and obstetric ultrasonography as per the hospital protocol. The patients who did not have spontaneous preterm birth were admitted by 37 to 37+6 weeks POG and were delivered by the suitable method as per institutional protocol. Subsequently, they were followed till 14 days post-delivery [7,8]. The feto-maternal outcome in the form of POG at the termination of pregnancy, onset of labor, mode of delivery, outcome of pregnancy, post-partum complication, APGAR score at 5th minute, birth weight, neonatal intensive care unit (NICU) admissions and other fetal and neonatal morbidity were recorded.

Data collection

As this was an ambispective study, the data of all retrospective cases from last year (January 2019 to January 2020) was retrieved from the medical records. From January 2020, all the patients attending ANC OPD and labor room who were diagnosed with IHCP were enrolled in the study.

Statistical analysis

For statistical analysis, the data were tabulated in an excel sheet and were analyzed by IBM Statistical Package for the Social Sciences (SPSS) for Windows, version 23.0. (IBM Corp, Armonk, New York, USA). Mean, range and standard deviation were used to describe the continuous variables, and percentages were used to describe the categorical data. The chi-square test or Fischer exact test was used to compare categorical data. The P-value was calculated by the Kruskal-Wallis test, value <0.05 was accepted as statistically significant. Spearman’s rank correlation coefficient was calculated for the definition of the strength of possible associations.

Ethical clearance

This study was started after approval of the Institutional Ethical Committee, AIIMS Jodhpur (AIIMS/IEC/2019/1067). The objectives and associated benefits of the study were explained to all the participants. The confidentiality of the patient’s details was maintained.

## Results

During the study period of two years, the total number of deliveries was 4,148. In this period a total of 152 patients were diagnosed with intrahepatic cholestasis of pregnancy. Out of these 152 singleton pregnant women, 140 (92.11%) had mild IHCP (BA 10-39 µmol/L), 10 (6.58%) had moderate IHCP (BA 40-99 µmol/L) and two (1.32%) had severe IHCP (BA ≥100 µmo/L) (Table 1).

**Table 1:** The maternal and perinatal demographic variables CPAP: Continuous positive airway pressure

Variables		Frequency (n)	Percentage (%)
Age (Years)	18-25	56	36.83
	26-30	58	38.16
	31-35	35	23.03
	>35	3	1.97
Period of gestation at diagnosis (Weeks)	28-31.6	12	7.89
	32-36.6	107	70.39
	37-39.6	32	21.05
	>= 40	1	0.66
Total bilirubin (mg/L)	<1	141	92.76
	>=1	11	7.24
Alanine aminotransferase (IU/L)	<40	32	21.05
	>=40	120	78.95
Aspartate aminotransferase (IU/L)	<40	27	17.76
	>=40	125	82.24
Alkaline phosphatase (IU/L)	<150	29	19.08
	>=150	123	80.92
Severity of Intrahepatic Cholestasis of pregnancy	Mild	140	92.11
	Moderate	10	6.58
	Severe	2	1.32
Treatment with Ursodeoxycholic acid		113	74.34
Associated medical disorder	Gestational diabetes mellitus	14	9.22
	Hypertensive disorder of pregnancy	17	11,19
	Thyroid disorder	22	14.48
Period of gestation at the termination of pregnancy (weeks)	28-31.6	3	1.97
	32-36.6	21	13.82
	37-39.6	125	82.24
	>=40 weeks	3	1.97
Onset of labor	Induced at term	92	60.53
	Spontaneous (Term & Preterm)	49	32.24
	Induced Preterm labor	11	7.24
Mode of delivery	Cesarean Section	48	31.58
	Normal vaginal delivery	104	68.42
PER Delivery finding	Meconium stained liquor	19	12.5
	Cord around neck	3	1.97
	None	130	85.53
APGAR at 5 min	Less than 7	2	1.33
	More than or equal to 7	148	98.67
Birth Weight	Less than 1500 gm	2	1.32
	1500-2500 gm	22	14.47
	More than 2500 gm	128	84.21
Neonatal Outcome	Stillbirth	2	1.32
	CPAP*	10	6.58
	Uneventful	140	92.11

The average age of pregnant women affected by IHCP was 27.63±3.91. A large number of pregnant women (74.34%) were treated with tablet ursodeoxycholic acid (UDCA) 10-15 mg/kg in divided doses as per severity. About 25.65% (n=29) of patients reported at 37 weeks period of gestation who underwent termination of pregnancy immediately after the diagnosis. As far as the medical disorder is concerned, only 14.48% of patients had hypothyroidism, 11.19% developed the hypertensive disorder during pregnancy, and 9.22% of pregnant women developed gestational diabetes mellitus but none had a history of IHCP in the previous pregnancy.

In the present study, two (1.32%) patients with moderate IHCP had intrauterine fetal death in the third trimester, 6.58% (n=10) and 92.10% (n=140) neonates were kept on continuous positive airway pressure and had uneventful neonatal outcomes respectively (Table 2).

**Table 2:** Maternal demography and laboratory variables according to the severity of IHCP Values are presented as median (interquartile range); “A” Significant difference between mild and moderate levels; “B” Significant difference between moderate and severe levels; “C” Significant difference between mild and severe levels; P value by Kruskal-Wallis test. IHCP: Intrahepatic cholestasis of pregnancy

Maternal Demography		Mild IHCP (n=140)	Moderate IHCP (n=10)	Severe IHCP (n=02)	P-value
Age (Years)	18-20	3 (75%)	1 (25%)	0 (0%)	0.44
	21-25	50 (96.15%)	2 (3.85%)	0 (0%)	
	26-30	53 (91.38%)	4 (6.9%)	1 (1.72%)	
	31-35	32 (91.43%)	2 (5.71%)	1 (2.86%)	
	>35	2 (66.67%)	1 (33.33%)	0 (0%)	
Gravida	Primi	53 (92.98%)	3 (5.26%)	1 (1.75%)	0.82
	Multi	87 (91.58%)	7 (7.37%)	1 (1.05%)	
Haemoglobin (gm%)		10.5 (9.6-11.7)	11.31 (11-11.9)	10.8 (10.2-11.4)	0.18
Liver function test	Total Bilirubin (mg%)	0.65 (0.425-0.79)	1.1 (0.9025-1.5)	0.95 (0.915-0.985)	<0.001^ A^
	SGPT (IU/L)	81 (43.75-100)	167 (90.5-238.75)	229 (174.5-283.5)	0.005 ^ABC^
	SGOT (IU/L)	78 (44-103.25)	139 (79.25-197.25)	186 (152-220)	0.02 ^A^
	ALP (IU/L)	304.5 (168-416)	328.5 (156.25-395.25)	316.5 (246.75-386.25)	0.95

There was no significant association of maternal age, gravida and hemoglobin levels with the severity of IHCP. However, there was a significant elevation of aspartate transaminase (AST), alanine transaminase (ALT) and total bilirubin levels related to the severity of IHCP (P-value <0.001). The period of gestation at the time of diagnosis of IHCP was significantly lower in mild and moderate IHCP (P-value 0.028). Nearly 16% of pregnant women with IHCP with a period of gestation less than 37 weeks underwent the termination of pregnancy, out of this 1.97% (n=3) had termination of pregnancy in less than 32 weeks of POG and this difference was also statistically significant (P-value 0.0006).

Labor was induced in 92 (60.53%) patients and 32.24% (n=49) pregnant women who underwent spontaneous term or preterm (PT) labor. In the study, the average weight of the baby was 2652 gms. There was a significant difference between the birth of the baby in mild, moderate and severe IHCP (P-value 0.004) but the APGAR score was comparable in the groups at 1 minute and 5 minutes 0.20 and 0.66 respectively (Table 3).

**Table 3:** Feto-maternal outcome according to the severity of IHCP Values are presented as median (interquartile range); “A” Significant difference between mild and moderate levels; “B” Significant difference between moderate and severe levels; “C” Significant difference between mild and severe levels; P-value by Kruskal-Wallis test. IHCP: Intrahepatic cholestasis of pregnancy; LSCS: Lower section cesarean section; POG: Period of gestation; CPAP: Continuous positive airway pressure.

Feto-Maternal Outcome		Mild IHCP (n=140)	Moderate IHCP (n=10)	Sever IHCP (n=02)	P-value
POG at Diagnosis of IHCP		35 (33.5-36.55)	32.85 (32-34.45)	29.3 (28.95-29.65)	0.028 ^A^
POG at Termination of pregnancy	28-31.6 weeks	1 (33.33%)	2 (66.67%)	0 (0%)	0.0006
	32-36.6 weeks	17 (80.95%)	3 (14.29%)	1 (4.76%)	
	37-39.6 weeks	119 (95.2%)	5 (4%)	1 (0.8%)	
	>=40 weeks	3 (100%)	0 (0%)	0 (0%)	
Mode of Labor	Induced at term	86 (93.48%)	5 (5.43%)	1 (1.09%)	0.92
	Spontaneous (Term & PT)	44 (89.8%)	4 (8.16%)	1 (2.04%)	
	Induced at Preterm	10 (90.91%)	1 (9.09%)	0 (0%)	
Mode of delivery	Vaginal delivery	95 (91.35%)	7 (6.73%)	2 (1.92%)	0.33
	LSCS	45 (93.75%)	3 (6.25%)	0 (0%)	
APGAR score	1 minute	8 (8-9)	8 (7.25-8)	4 (2-6)	0.2
	5 minute	9 (9-10)	9 (9-9.75)	9 (9-9)	0.66
Weight of baby		2865 (2705-3140)	2315 (2207.5-2685)	1525 (1287.5-1762.5)	0.004 ^ABC^
Neonatal outcome	Uneventful	131 (93.57%)	7 (5%)	2 (1.43%)	<0.0001
	CPAP	9 (90%)	1 (10%)	0 (0%)	
	Intrauterine death	0 (0%)	2 (100%)	0 (0%)	

Total 12.5% (n=19) patients had meconium-stained liquor during delivery, intrauterine death occurred in 1.31% (n=2) moderate IHCP cases and about 85% (n=130) pregnant women with IHCP had uneventful delivery. This difference was statically significant in neonatal outcomes (P-value-0.001).

The correlation of level of liver function variables with bile acid levels is given in Table 4.

**Table 4:** Correlation of ‘r’ between maternal serum bile acid and variables of Liver function test

Liver function test	Serum Bile acid	P-value
Total Bilirubin	r=0.48	<0.001 HS
SGPT	r=0.54	<0.001 HS
SGOT	r=0.48	<0.001 HS
ALP	r=0.10	0.22 NS

**Figure 1:**
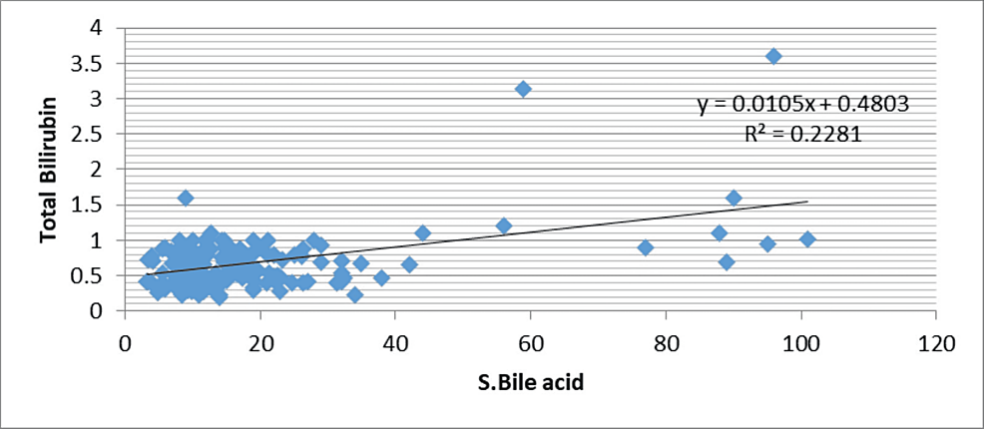
Correlation of maternal serum bile acid levels with total bilirubin

**Figure 2:**
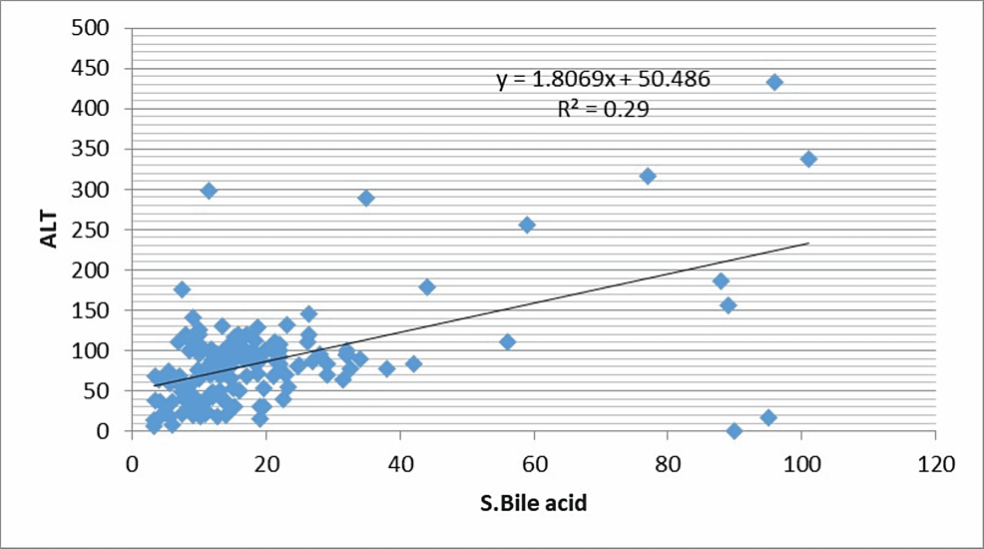
Correlation of maternal serum bile acid levels with alanine aminotransferase

**Figure 3:**
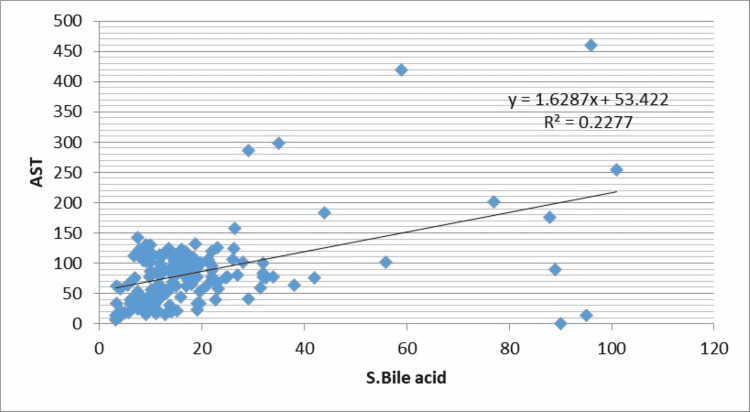
Correlation of maternal serum bile acid levels with aspartate aminotransferase

**Figure 4:**
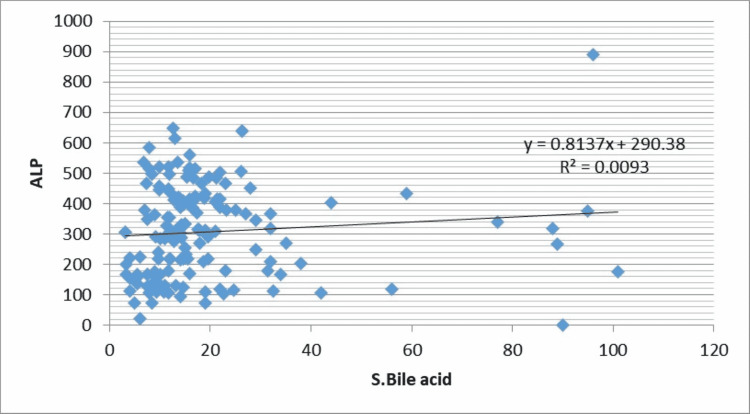
Correlation of maternal serum bile acid levels with alkaline phosphatase

As shown in Table 3 and Figure 1, there is significant positive correlation of maternal serum bile acid with total bilirubin (r=0.48, P-value=<0.001), alanine aminotransferase (r=0.54, P-value-<0.001), aspartate aminotransferase (r=0.48, P-value-<0.001) but weak correlation was seen between serum bile acid with alkaline phosphatase (r=0.10, P-value-0.22) (Figures 1-4).

## Discussion

This is the first large study showing the correlation of variables of liver function test with maternal serum bile acid along with the effect of intrahepatic cholestasis of pregnancy on the fetomaternal outcome.

In the present study, 152 pregnant women with IHCP were enrolled and were divided into three groups according to their bile acid levels. About 58% of pregnant women with moderate IHCP (BA 40-99 µmol/L) were from the 26-30 years age group but there was no significant correlation between maternal age and IHCP which is supported by the study conducted by Shukla et al. [9].

The present study revealed that the incidence of IHCP was about 62.5% in multigravida and 37.5% in primigravidae. However, there was no significant difference between the groups. Pillarisetty and Sharma did not find any association between IHCP and multigravida but concluded an increased risk of IHCP in a multifetal pregnancy [10].

There are a few limitations to getting serum bile acid tested like the non-availability of the test kit and the cost. The liver function test can be easily done at any center and it is more cost-effective than the total bile acid test. In the present study, we have found a good correlation between serum bile acid level and variables of liver function test. Hence, if the patient is not able to afford the bile acid test we can follow the pregnancy with a liver function test on a weekly basis. Our findings were supported by Wang et al. who concluded that in cholestasis of pregnancy the ALT ≥ 50 U/L is associated with BA ≥10 µmol/L and it can be useful in the diagnosis and management of IHCP if the total bile acid test is not available [11].

Ursodeoxycholic acid is the preferred pharmacological drug for the treatment of IHCP, it helps in the reduction of symptoms, levels of serum bile acid and adverse perinatal outcomes [12]. In the present study, 74.34% of patients with IHCP received ursodeoxycholic acid, rest 25.66% of patients underwent termination of pregnancy as they reported beyond 37 weeks of POG.

Almost 70.40% (n=107) of pregnant women were diagnosed with IHCP at 32-36.6 weeks of pregnancy. In the present study, 15.78% (n=24) of pregnant women had a preterm delivery as opposed to a national preterm birth rate of 8-12% [9].

Among these, nearly 16% of pregnant women with IHCP had a termination of pregnancy with a period of gestation less than 37 weeks underwent, out of this 1.97% (n=3) had a termination of pregnancy in less than 32 weeks of POG. The reason behind the induction of labor at or less than 37 weeks of POG was deranged serum bile acid (BA 40-99 µmol/L). According to the Royal College of Obstetricians and Gynaecologists, clinicians should discuss the benefits and risks of induction of labor after 37 weeks of gestation with women affected by ICP to prevent the adverse perinatal outcome [13].

Out of these 24 pregnant women, 41.66% (n=10) and 4.18% (n=1) were induced with mild IHCP and moderate IHCP respectively because they had associated medical disorders like preeclampsia and uncontrolled gestational diabetes mellitus. Nearly 13 (54.16%) pregnant women underwent spontaneous preterm labor, which is thought to be occurred due to increased expression and sensitivity of oxytocin receptors in the myometrium [14].

In this study, the rate of emergency lower section cesarean section (LSCS) was near about 50% of vaginal delivery in mild and moderate IHCP, however, the difference was statistically not significant. About 12.5% of laboring patients had meconium-stained liquor leading to fetal distress; to provide standard antenatal care and to prevent adverse perinatal outcomes the decision of emergency LSCS was taken which was also advocated by Puljic et al. [15].

It was noticed that the pregnancy complicated by IHCP affects the fetomaternal outcome significantly in terms of the period of gestation at the termination of pregnancy, meconium-stained liquor, fetal distress, low APGAR score, the weight of baby and NICU admission and intrauterine fetal death which is also confirmed by the study conducted by Brouwers at al. [16].

The average weight of the baby was 2652 gms. There is a significant difference (P-value-0.004) in the birth weight of newborns among all three groups which can be justified by spontaneous preterm labor in IHCP patients and iatrogenic induction of labor to prevent adverse perinatal outcomes. A similar finding was found in studies conducted by Posh et al. and Li et al.; 40% of newborns had a birth weight less than 2500 gms and neonatal birth weight was lower in pregnancy complicated by IHCP than in low-risk pregnancies (weighted mean difference: 267 gm, 95% CI) [17, 18].

In IHCP, the risk of stillbirth varies from 0.4% to 7% [3, 17]. The chances of stillbirth are more common at term after 37 weeks of period of gestation and are associated with bile acid 100 µmol/L or more. In present study, 1.32% (n=2) pregnant women had stillbirth, 6.58% (n=10) babies were kept on continuous positive airway pressure (CPAP) and 92.11% (n=140) had uneventful neonatal outcome. It implies that pregnant women with moderate or severe IHCP warrant strict fetomaternal surveillance to prevent adverse perinatal outcomes.

Strength

To the best of my knowledge, this is the first study on IHCP showing a correlation of serum bile acid with variables of liver function test. No other study has been done to find out the correlation of serum bile acid with variables of liver function test.

Limitations

The limitation of this study is the lack of control data to compare the perinatal outcome like meconium-stained liquor, APGAR score, birth weight, and the requirement of CPAP.

## Conclusions

Intrahepatic cholestasis of pregnancy is associated with adverse feto-maternal outcomes. Biochemically, IHCP is characterized by raised serum bile acid and raised aminotransferase with or without hyperbilirubinemia. In the present study, there is a significant correlation between raised bile acid levels and variables of liver function test in IHCP. As many patients can’t afford to get serum bile acid done for follow-up of IHCP, LFT can be used as a good alternative to serum bile acid. The liver function test is feasible, easily assessable and cost-effective to diagnose and for follow-up of pregnant patients with IHCP. Hence liver function test can be considered as an alternative to bile acids.
